# Intracerebral Iron Accumulation may be Associated with Secondary Brain Injury in Patients with Poor Grade Subarachnoid Hemorrhage

**DOI:** 10.1007/s12028-021-01278-1

**Published:** 2021-08-09

**Authors:** Raimund Helbok, Verena Rass, Mario Kofler, Heribert Talasz, Alois Schiefecker, Max Gaasch, Christoph Scherfler, Bettina Pfausler, Claudius Thomé, Ronny Beer, Herbert H. Lindner, Erich Schmutzhard

**Affiliations:** 1 Neurological Intensive Care Unit, Department of Neurology, Innsbruck Medical University, Innsbruck, Austria; 2 Division of Clinical Biochemistry, Biocenter, Innsbruck Medical University, Innsbruck, Austria; 3 Department of Neurosurgery, Innsbruck Medical University, Innsbruck, Austria

**Keywords:** Cerebral microdialysis, Subarachnoid hemorrhage, Iron, Multimodal neuromonitoring, Neurocritical care

## Abstract

**Background:**

The amount of intracranial blood is a strong predictor of poor outcome after subarachnoid hemorrhage (SAH). Here, we aimed to measure iron concentrations in the cerebral white matter, using the cerebral microdialysis (CMD) technique, and to associate iron levels with the local metabolic profile, complications, and functional outcome.

**Methods:**

For the observational cohort study, 36 patients with consecutive poor grade SAH (Hunt & Hess grade of 4 or 5, Glasgow Coma Scale Score ≤ 8) undergoing multimodal neuromonitoring were analyzed for brain metabolic changes, including CMD iron levels quantified by graphite furnace atomic absorption spectrometry. The study time encompassed 14 days after admission. Statistical analysis was performed using generalized estimating equations.

**Results:**

Patients were admitted in a poor clinical grade (*n* = 26, 72%) or deteriorated within 24 h (*n* = 10, 28%). The median blood volume in the subarachnoid space was high (SAH sum score = 26, interquartile range 20–28). Initial CMD iron was 44 µg/L (25–65 µg/L), which significantly decreased to a level of 25 µg/L (14–30 µg/L) at day 4 and then constantly increased over the remaining neuromonitoring days (*p* < 0.01). A higher intraventricular hemorrhage sum score (≥ 5) was associated with higher CMD iron levels (Wald-statistic = 4.1, df  = 1, *p* = 0.04) but not with the hemorrhage load in the subarachnoid space (*p* = 0.8). In patients developing vasospasm, the CMD iron load was higher, compared with patients without vasospasm (Wald-statistic = 4.1, degree of freedom = 1, *p* = 0.04), which was not true for delayed cerebral infarction (*p* = 0.4). Higher iron concentrations in the brain extracellular fluid (34 µg/L, 36–56 µg/L vs. 23 µg/L, 15–37 µg/L) were associated with mitochondrial dysfunction (CMD lactate to pyruvate ratio > 30 and CMD-pyruvate > 70 µM/L, *p* < 0.001). Brain extracellular iron load was not associated with functional outcome after 3 months (*p* > 0.5).

**Conclusions:**

This study suggests that iron accumulates in the cerebral white matter in patients with poor grade SAH. These findings may support trials aiming to scavenger brain extracellular iron based on the hypothesis that iron-mediated neurotoxicity may contribute to acute and secondary brain injury following SAH.

## Introduction

Aneurysmal subarachnoid hemorrhage (aSAH) is still a devastating disease associated with a high mortality and leads to a substantial long-term morbidity. It is well known that the amount of blood released to the subarachnoid and intraventricular space at ictus correlates with early neurological worsening, increased rates of hospital complications including delayed cerebral ischemia (DCI), and poor functional outcome [1–4]. Attempts to decrease the hemorrhage load by intracisternal clot removal and ventricular or lumbar drainage effectively reduced the risk of DCI; however, they failed to translate into improved long-term outcomes [5–7]. A recent retrospective trial investigating the combined approach of aggressive clot removal and continuous intravenous nicardipine administration showed promising results but needs to be confirmed in a prospective multicenter trial [8]. Moreover, the results of the recently terminated trial that investigates the effect of lumbar drainage on 6-month functional outcome are still pending EARLYdrain - Outcome After Early Lumbar CSF-drainage in Aneurysmal SAH (EARLYDRAIN), NCT01258257 [9].

There is a need to further elaborate on pathophysiologic mechanisms related to the hemorrhage load that could potentially serve as a treatment target in future trials. It is well known that erythrocytes lyse in the subarachnoid and intraventricular space after SAH and expose the brain to hemoglobin and its degradation product heme, which are capable of producing free radicals if not neutralized, i.e., by haptoglobin and hemopexin, and taken up by macrophages [10, 11]. Heme is then converted into carbon monoxide, biliverdin, and iron by the heme oxygenase [12, 13]. Evidence suggests that iron overload can contribute to brain damage after intracerebral hemorrhage [14]. In a small series including 12 patients with SAH, increased iron levels in the cerebrospinal fluid were associated with DCI [15]. Recently, we could show that iron is trapped throughout large portions of the cerebral white matter after SAH using statistical parametric mapping analysis of the R2* signal on sequential magnetic resonance imaging [16]. Interestingly, iron accumulation was already observed three weeks after the bleeding and remained elevated at the 1-year follow-up imaging. Moreover, we found that axonal damage colocalized with iron accumulation and was associated with neuropsychological deficits [16]. So far, direct measurement of iron load in the brain tissue after SAH is limited but may be feasible by analyzing the brain extracellular fluid of patients monitored with cerebral microdialysis (CMD). CMD allows online measurement of brain extracellular metabolites for clinical use and research [17].

Based on previous studies showing iron accumulation after SAH, the goal of the current study was to quantify daily iron levels in the brain extracellular fluid and relate these findings to the intracranial hemorrhage load, brain metabolism, hospital complications (e.g., vasospasm and DCI), and functional outcome in patients with poor grade SAH. We hypothesized that a higher load of CMD iron, including free iron as well as iron bound in hemoglobin, heme, or the heme–hemopexin complex, is associated with local metabolic abnormalities, hospital complications, and poor functional outcome.

## Methods

The data that support the findings of this study are available from the corresponding author on reasonable request.

### Study Design, Setting, and Patient Selection

**Fig. 1:**
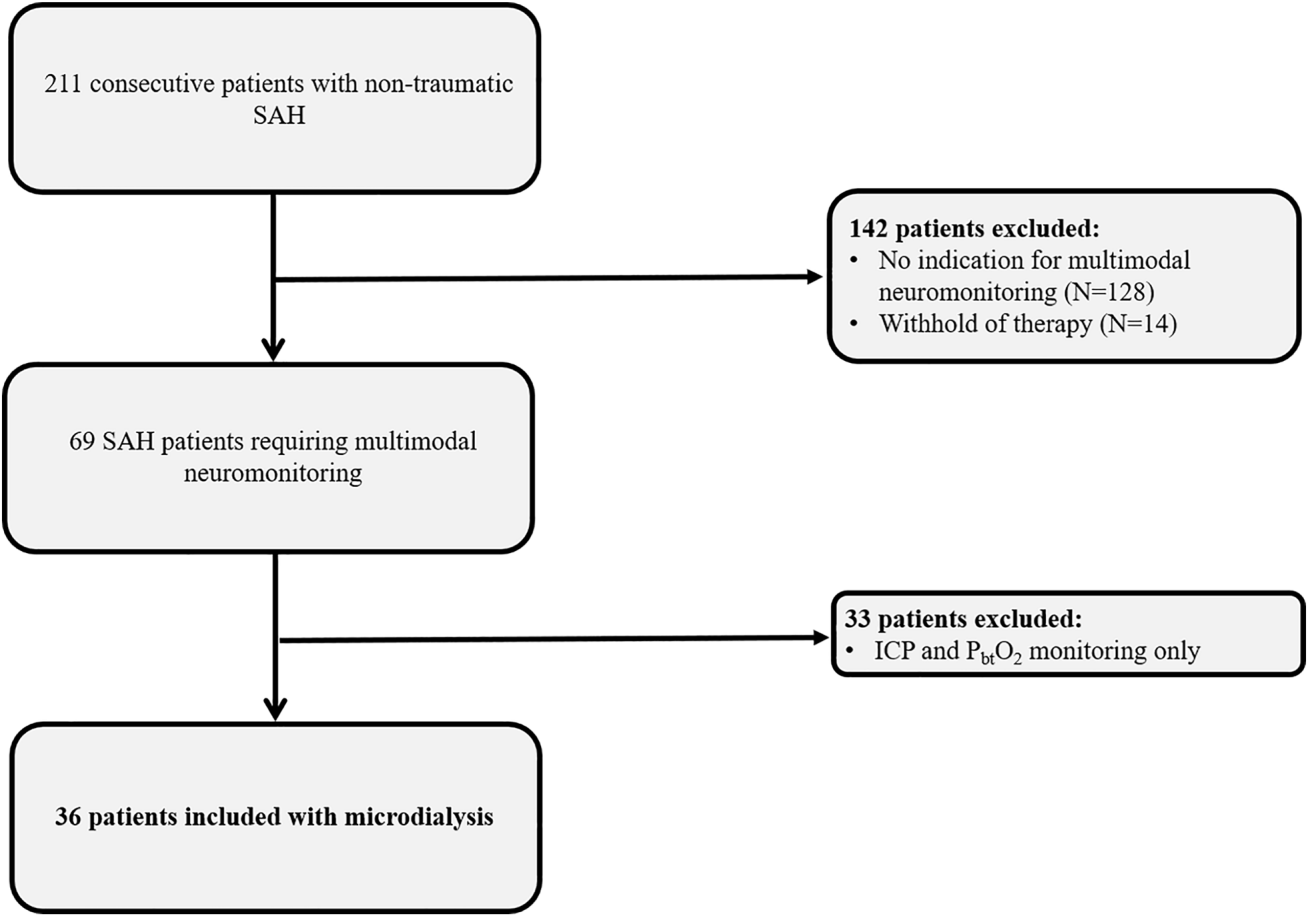
Flow chart showing patient selection. *ICP* Intracranial pressure, *P*_*bt*_*O*_*2*_ Brain tissue oxygen tension, *SAH* Subarachnoid hemorrhage

The study design was guided by the STROBE statement on observational cohort studies. Between 2010 and 2015, 36 patients with consecutive poor grade aSAH admitted to the neurological intensive care unit at the Innsbruck Medical University were prospectively included for retrospective data analysis. General inclusion criteria encompassed (1) spontaneous aSAH, (2) age ≥ 18 years, and (3) multimodal neuromonitoring including CMD as part of routine clinical care (Fig. 1). Invasive multimodal neuromonitoring was initiated in patients with poor grade SAH with depressed consciousness at presentation (Glasgow Coma Scale score ≤ 8) or early neurological worsening and in those with expected prolonged mechanical ventilation and/or clinical or radiologic signs suggestive of increased intracranial pressure according to the local institutional protocol. The conduct of the study was approved by the ethics committee of the University of Innsbruck, which is in compliance with the Helsinki Declaration (AN3898 285/4.8, AM4091-292/4.6). Written informed consent was obtained according to federal regulations.

### Patient Care

The clinical care of patients with aSAH conforms to guidelines set forth by the American Heart Association [18]. All patients received a cerebral angiogram to confirm the diagnosis. The treatment modality (clipping or coiling) of the ruptured aneurysm was decided in a multidisciplinary discussion (neurosurgery, neurology, and neuroradiology) based on aneurysm growth, localization, and patient age. All patients were regularly followed with transcranial color-coded duplex sonography (TCD, LOGIQ S8; GE Healthcare, Chicago, IL) and received continuous intravenous nimodipine. Acceleration of TCD mean blood flow velocities (mBFV) > 120 cm/s in the middle or anterior cerebral artery or a daily change in mean TCD-velocities greater than 50 cm/s was suggestive of large-vessel cerebral vasospasm. A catheter cerebral angiogram was performed in patients with severe vasospasm (TCD-mBFV > 200 cm/s) refractory to hypertensive therapy [cerebral perfusion pressure (CPP) target > 80 mm Hg]. These patients were treated with intraarterial nimodipine. Cerebral infarction from DCI was defined as the appearance of a new infarction on head computed tomography (CT) scan that was judged by an independent radiologist not to be attributed to other causes [19]. All patients were comatose and treated with continuous sufentanil and/or ketamine and midazolam drips to facilitate mechanical ventilation.

### Grading of the Intracranial Hemorrhage Load

The intracranial hemorrhage volume was quantified in the subarachnoid and intraventricular space using the SAH sum score, which grades the amount of blood in ten basal cisterns and fissures (0 = no SAH, 1 = small SAH, 2 = moderate SAH, 3 = completely filled with SAH) by adding each of the ten individual cistern scores (range 0–30), and the intraventricular hemorrhage (IVH) sum score, which grades the amount of blood in the right and left lateral, third and fourth ventricle (0 = no blood, 1 = sedimentation, 2 = partly filled, 3 = completely filled) by adding each of the four individual ventricle scores (range 0–12).

### Data Collection and Neuromonitoring

All admission variables and hospital complications were prospectively recorded in our institutional SAH outcome database. Functional outcome was assessed by a study nurse blinded to the clinical course of patients 3 months post bleeding using the modified Rankin Scale (mRS) with poor outcome defined as mRS > 2.

Through a burr hole, a triple-lumen bolt was affixed to insert an ICP parenchymal probe (Neurovent_P-Temp; Raumedic, Münchberg, Germany). In addition, a high cutoff brain microdialysis catheter (CMA-71, pore size 100 kDa; M-Dialysis, Stockholm, Sweden) was tunneled and inserted into the brain parenchyma for hourly assessment of brain metabolism. Isotonic perfusion fluid (Perfusion Fluid CNS; M-Dialysis) was pumped through the system at a flow rate of 0.3 µl/minute. Hourly samples were analyzed with CMA 600 and Iscus^flex^ (M-Dialysis*)* for cerebral extracellular glucose, pyruvate, lactate, and glutamate concentrations. At least 1 h passed after the insertion of the probe and the start of the sampling to allow for normalization of changes due to probe insertion. After routine analysis, samples were kept at − 80 °C. Monitoring devices were inserted into the parenchyma of the vascular territory of the parent vessel of the aneurysm and the location confirmed by brain CT immediately after the procedure and classified as placed in morphologically “normal” tissue or “perilesional” (< 1 cm distant from the lesion) [20]. Brain metabolic distress was defined as lactate to pyruvate ratio (LPR) > 40 [21] and mitochondrial dysfunction as LPR > 30 together with a CMD-pyruvate > 70 μM/L [22]. All continuously measured parameters were saved on a 3-min average interval using our patient data management system (CentricityTM Critical Care 8.1 SP2; GE Healthcare Information Technology, Dornstadt, Germany).

### Analytical Methods

Cerebral microdialysis iron was quantified by graphite furnace atomic absorption spectrometry (M6 Zeeman GFAA-Spectrometer; Thermo Scientific) at 248.3 nm and Zeeman background correction using 1000 °C ash temperature and 2100 °C atomization temperature under argon atmosphere [23]. Using this method, both free iron as well as bound iron as part of hemoglobin, heme, or the heme–hemopexin complex is measured. Because of the pore size of the CMD membrane, we expected that hemoglobin (65 kD), haptoglobin (86 kD), heme (0.616 kDa), hemopexin (60 kD), iron, and the hemopexin-heme complex could pass the membrane but not the haptoglobin–hemoglobin complex involved in the hemoglobin scavenging pathway of extravascular hemolysis.

### Statistical Analysis

Continuous variables were assessed for normality and reported as mean ± standard error of mean (SEM) or median and interquartile range (IQR). Categorical variables were reported as counts and proportions in each group. Hourly recorded concentrations in the cerebral microdialysate were matched to continuously recorded parameters (ICP, CPP) averaged over the sampling period within the study period of 14 days after admission. CMD-metabolic parameters were categorized as previously defined according to international accepted definitions to associate with CMD iron levels [24]. Correlation levels between CMD iron levels and metabolic parameters were assessed with use of the Pearson correlation coefficient. Time series data were analyzed with generalized linear models using a normal distribution and identity-link function and were extended by generalized estimating equations with an autoregressive process of the first order to handle repeated observations within a patient [25]. Data were transformed (log for CMD iron) to meet assumptions of normality. All models were adjusted for disease severity (H&H grade), age, and the time of CMD collection relative to the bleeding and laboratory analysis. For all tests, the significance level was set at *p* < 0.05. All analyses were performed with IBM-SPSS V20.0 (IBM Corporation, Chicago, IL).

## Results

### General Characteristics

Thirty-six patients met the inclusion criteria. Clinical characteristics, hospital complications, and outcome data are summarized in Table 1. Most patients were admitted in a poor clinical grade (Hunt & Hess grade 4 and 5; *n* = 26, 72%) or deteriorated within 24 h (*n* = 10, 28%). The aneurysm was secured within 5 h (2–14 h) after diagnosis by endovascular coiling (*n* = 15, 42%) or surgical clipping (*n* = 21, 58%). The median blood volume in the subarachnoid space was high (SAH sum score = 26, IQR 20–28), and nine patients developed DCI (25%). The multimodal neuromonitoring bundle was inserted at a median of 11 (4–26) hours after admission to the ICU. In nine patients (25%) CMD catheters were located perilesional and in all other patients in normal appearing brain tissue. Three patients died during hospitalization (8%).

**Table 1:** Baseline characteristics, complications, and outcomes

		*N* = 36
*Clinical characteristics*		
Age (year)		57 (48–67)
Female sex		26 (72%)
Admission Hunt & Hess grade	2 3 4 5	3 (8%) 7 (19%) 3 (8%) 23 (64%)
Loss of consciousness at ictus		20 (56%)
*Admission radiological characteristics*		
Modified Fisher scale	2 3 4	3 (8%) 16 (44%) 17 (47%)
SAH sum score		26 (20–28)
IVH sum score		5 (2–6)
Aneurysm size above 10 mm		9 (25%)
Generalized cerebral edema		15 (42%)
Co-occurrence of intraparenchymal hematoma		12 (33%)
*Surgical procedures*		
Hydrocephalus requiring EVD		26 (72%)
Clipping		21 (58%)
Hemicraniectomy		5 (14%)
*Complications*		
Pneumonia		25 (69%)
Ventriculitis		4 (11%)
Vasospasm		24 (67%)
Delayed cerebral infarction		9 (25%)
Anemia requiring transfusion		20 (56%)
Aneurysm rebleeding		5 (14%)
*Outcome characteristics*		
Length of hospital stay (d)		30 (23–44)
3-month modified Rankin Scale Score	0 1 2 3 4 5 6	1 (3%) 8 (22%) 3 (8%) 7 (19%) 4 (11%) 8 (22%) 5 (14%)

Data are given in median (IQR) and counts (%). SAH sum score grades the amount of blood in ten basal cisterns and fissures (0 = no SAH, 1 = small SAH, 2 = moderate SAH, 3 = completely filled with SAH) by adding each of the ten individual cistern scores (range 0–30); IVH sum score grades the amount of blood in the right and left lateral, third and fourth ventricle (0 = no blood, 1 = sedimentation, 2 = partly filled, 3 = completely filled) by adding each of the four individual ventricle scores (range 0–12)

*EVD* External ventricular drain, *IQR* Interquartile range, *IVH* Intraventricular hemorrhage, *SAH* Subarachnoid hemorrhage

### Brain Extracellular Iron

**Fig. 2:**
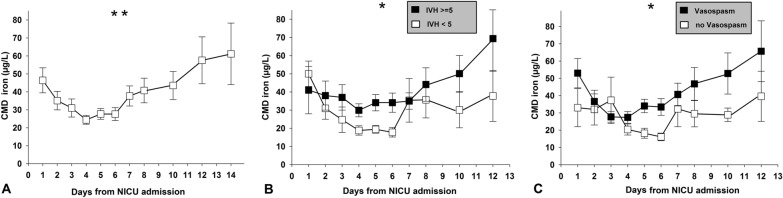
Daily mean brain extracellular iron levels of 36 patients with aSAH (mean ± SEM). Panel **a** shows longitudinal concentrations over days after SAH, with panels **b** and **c** indicating groups with and without significant IVH and large-vessel vasospasm. *p < 0.05, **p < 0.01. *aSAH* Aneurysmal subarachnoid hemorrhage, *CMD* Cerebral microdialysis, *IVH* Intraventricular hemorrhage, *NICU* Neurological intensive care unit, *SAH* Subarachnoid hemorrhage, *SEM* Standard error of the mean

Initial CMD iron was 44 µg/L (25–65 µg/L), significantly decreased to a level of 25 µg/L (14–30 µg/L) at day 4 and constantly increased over the remaining neuromonitoring days (Fig. 2a, *p* < 0.01). Overall CMD iron was not different in patients with perilesional microdialysis catheter placement (27 µg/L, 20–40 µg/L) when compared with patients with catheters placed into normal appearing brain tissue on head CT-scan (27 µg/L, 21–41 µg/L). Intracerebral hematoma was equally distributed among both hemispheres and distant from the monitoring probes. Still, the blood load in the subarachnoid space was higher in the monitored hemisphere. In addition, there was no difference in iron levels in patients with or without hydrocephalus requiring EVD, even after controlling for IVH (*p* = 0.4).

A higher intraventricular hemorrhage sum score (≥ 5) was associated with higher CMD iron levels (Wald-statistic = 4.1, degree of freedom = 1, *p* = 0.04; Fig. 2b) but not the hemorrhage load in the subarachnoid space, quantified by the SAH sum score (*p* = 0.8). In patients developing vasospasm, CMD iron load was higher compared with those where repeated TCD never indicated large-vessel vasospasm (Fig. 2c; Wald-statistic = 4.1, degree of freedom = 1, *p* = 0.04). No difference in CMD iron was found in patients with and without DCI (*p* = 0.4).

Fifty-six percent of patients developed profound anemia requiring at least one red blood cell transfusion, which was not associated with a higher brain iron load (*p* > 0.5). Brain extracellular iron load was not associated with functional outcome after 3 months (*p* > 0.5).

### Brain Extracellular Iron and Other Microdialysis Analytes

There was a significant correlation between CMD iron and CMD LPR (*r* = 0.38, *p* < 0.001), and a weaker correlation with CMD-lactate (*r* = 0.27, *p* < 0.001), CMD-glutamate (*r* = 0.26, *p* < 0.001), and CMD-glucose (*r* =  − 0.15, *p* = 0.02). Higher CMD iron concentrations (40 µg/L, 28–63 µg/L vs. 24 µg/L, 15–38 µg/L; *p* < 0.001) were observed during episodes of brain metabolic distress (CMD LPR > 40), which was particularly true for mitochondrial dysfunction in the presence of normal or high pyruvate levels (> 70 µM/L; 34 µg/L, 36–56 µg/L vs. 23 µg/L, 15–37 µg/L, p < 0.001) but not ischemia (pyruvate < 70 µM/L; 30 µg/L, 27–63 µg/L vs. 28 µg/L, 17–44 µg/L).

## Discussion

The cardinal findings of this study are that (1) iron can be quantified in the brain extracellular fluid of patients with poor grade SAH, (2) iron levels are elevated in the white matter remotely from the bleeding source and constantly increase over time after SAH, (3) iron concentrations correlate with the IVH load, and (4) are higher in patients with brain mitochondrial dysfunction, and in those who develop large-vessel cerebral vasospasm. Importantly, all models were adjusted for disease severity (H&H grade) and age. These findings together with our previous report [16] suggest that iron accumulates in the white matter of the brain already in the acute phase after SAH, is associated with mechanisms of secondary brain injury, and may be trapped in the brain tissue for a prolonged time after SAH.

Following SAH, the brain is exposed to high concentrations of hemoglobin and its downstream products not only on the surface of the brain, but also in deeper layers of the cortex. Although iron is an essential cofactor for myelination, synthesis of neurotransmitters and neuronal development, free iron can lead to oxidative stress and neuronal cell damage through radical formation secondary to Fenton reactions [26, 27]. Iron is transported into the brain extracellular fluid Under healthy conditions, through receptor-mediated endocytosis of the brain capillary endothelial cells and subsequently, which is less clear, into the brain extracellular fluid where it is found unbound and transferrin-bound [28]. Iron is then taken up by neurons, astrocytes and oligodendrocytes [28]. Transport mechanisms in the injured brain are less clear. Interestingly, the second increase of CMD iron in our study was more pronounced in patients with higher intraventricular blood load. One potential explanation is that iron may diffuse from the ventricular system to the white matter of the brain. It is well known that the inner CSF-brain barrier at the level of the ventricular wall is discontinuous and literally nonexistent in adults [29] which may support our hypothesis. Although we cannot proof causality, this hypothesis seems intriguing and needs further confirmation.

In a previous study, we found progressive axonal damage after 12 months, compared with three weeks magnetic resonance imaging in patients with good grade SAH that colocalized with iron accumulation [16]. Although structural damage of axons and neurons may be multifactorial after SAH, neuronal iron accumulation has been linked to chronic neurodegenerative disorders such as Parkinson disease and Alzheimer disease [30, 31]. It is important to notice that a causal association has never been proven in neurodegenerative diseases, and mechanisms of neuronal toxicity remain unclear [32]. Still our results are of interest in this context as we also found an association between iron accumulation and neuropsychological deficits 12 months after SAH [16]. Taking these results and our current finding, iron may accumulate already in the acute phase after the bleeding in the white matter of the brain distant to the aneurysm and then persist for a prolonged time in patients with SAH. Further research is needed with a prolonged follow-up period to understand the importance of this finding. We could not further elaborate on mechanisms of hemoglobin scavenging pathways including the hemopexin–CD91 scavenging system [33], or the CD163-haptoglobin–hemoglobin scavenging system [34], in the setting of extravascular hemolysis after SAH. We are well aware that our method is limited by the fact that we quantify the total iron content in the brain extracellular compartment, both bound and unbound iron. Therefore, we cannot conclude on the mechanism of the sequestering of hemoglobin and on toxic or neuroprotective effects of singular proteins. Accordingly, our study serves as a pilot study and further analyses elaborating on pathways of the hemoglobin scavenging system are necessary.

Interestingly, we found an association between higher extracellular iron concentrations and brain mitochondrial dysfunction. Cerebral mitochondrial dysfunction is common after SAH, and in our patients this specific metabolic profile was evident in 40% of neuromonitoring days. Although iron is essential for mitochondrial enzymes and therefore neuronal function, pathological increased levels are associated with mitochondrial damage and oxidative distress [35, 36]. In this line, experimental studies in SAH models suggest that iron overload can cause mitochondrial dysfunction in the brain and blockage of mitochondrial calcium uniporter may prevent iron accumulation and thus decrease reactive oxygen species (ROS) generation and brain injury [37]. Similarly, the association has been described in other neurodegenerative diseases and brain disorders of mitochondrial iron dys-homeostasis including Friedreich’s ataxia and sideroblastic anemia [36].

Longitudinal iron quantification over 14 days in our patients with SAH revealed an initial peak concentration decreasing to a minimum at day 4 followed by a constant increase thereafter. Manipulation of brain tissue by insertion of the microdialysis catheter (tip of the catheter used is around 130 μm in diameter) may cause disruption of the vascular architecture and the blood tissue barrier and therefore cause micro-bleeding along with biochemical changes which may explain higher iron levels in the initial phase [38]. This hypothesis is further supported by human data showing a proinflammatory response in the adjacent brain tissue surrounding the microdialysis probe [39]. The significance of this finding for clinical use seems negligible as the CMD catheter is still comparably small compared with the diameter of conventionally used ICP probes usually exceeding 1000 μm. For clinical metabolic monitoring it is recommended to discard the first hour of microdialysate collected, which was done in our patients [17].

The second increase in CMD iron concentrations was more pronounced in patients with significant intraventricular bleeding and patients who developed cerebral vasospasm. This finding is again supported by animal data showing a link between iron deposition and ROS formation, and human data underlining the role of hemoglobin and its degradation products as a key factor in the pathogenesis of cerebral vasospasm after SAH [15, 40, 41]. Interestingly, the delayed increase in iron levels was similar in patients with intraventricular IVH and vasospasm which support previous studies identifying higher intraventricular blood volume with increased risk of vasospasm and DCI [2]. Experimental data have shown that enhanced iron production colocalized with ROS formation in the subarachnoid space as pathogenic link to vasospasm and neuronal injury after SAH [40, 41]. In this line, higher iron levels in cerebrospinal fluid were associated with the development of vasospasm and DCI in a small series of 12 patients with SAH [15]. It is important to mention that we did not find an association of increased iron levels in the brain interstitium and DCI. However, the pathophysiologic mechanisms of DCI are well known to be multifactorial, including microthrombosis, endothelial dysfunction, neuroinflammation and cortical spreading depolarizations [42–45], and resolution of cerebral vasospasm has so far not been translated into improved neurological long-term outcome after SAH [46].

Treatment options to decrease the hemorrhage load and potentially influence iron accumulation after SAH remain unsatisfying so far [7]. A potential treatment option is to capture intracerebral iron with chelators. Deferoxamine has been tested safely in patients with intracerebral hemorrhage [47]. The “Deferoxamine in Aneurysmal Subarachnoid Hemorrhage Trial” is a Phase I-II trial investigating continuous deferoxamine administration over 3 days (ClinicalTrials.gov Identifier: NCT02875262). In experimental SAH, deferoxamine ameliorates brain injury by reduction of the brain nonheme iron concentration, iron-handling protein expression, oxidative stress, and neuronal cell death [48, 49]. In rodent intracerebral hemorrhage models, deferoxamine reduces hemoglobin‐induced brain edema and decreases brain injury [50, 51]. Importantly, deferoxamine can easily penetrate the blood barrier and accumulate in the brain tissue after systemic administration.

Our study was designed as a pilot study and included a small number of patients, which is a potentially limiting factor. Moreover, iron levels quantified in the current study may be relevant for patients with poor grade aSAH and not be generalizable to all clinical grades. In future studies, noninvasive tools, such as magnetic resonance imaging, are needed to quantify iron load in the white matter [16]. Spectrometric measurement of iron is not standardized to analyze the brain extracellular fluid. Still, the small variability of iron concentration measured over days suggests that longitudinal analysis over time is feasible and changes over time may be detected. Patients were selected for invasive neuromonitoring based on the clinical presentation and the likelihood for prolonged need for mechanical ventilation. Ten patients clinically deteriorated due to rebleeding (*n* = 5) or aggravation of global cerebral edema within 24 h. Importantly, we did not find differences in the brain iron profile in these patients compared with the patients who presented in poor clinical grade already on admission (*n* = 26; data not shown). We did not include a control group in our analysis. Importantly all patients enrolled were consecutive patients presenting to our ICU. Finally, we would like to emphasize the point that our results are primarily hypothesis generating and do not proof causality. Still, the results of this pilot study encourage to further study the hemoglobin scavenging system including the hemopexin–CD91 scavenging system, and the CD163-haptoglobin–hemoglobin scavenging system in patients with SAH.

## Conclusions

In this study, we show that brain extracellular iron can be measured in the microdialysate and is associated with mechanisms of secondary brain injury. Iron-mediated neurotoxicity following aneurysm rupture represents a promising therapeutic target that should be explored in future trials. Our data also raise concerns about local microhemorrhages caused by insertion of the microcatheter into the brain tissue. Still, these phenomena seem to be reversible but should be taken into account in the initial phase of monitoring.

## Abbreviations

aSAH: Aneurysmal subarachnoid hemorrhage
CMD: Cerebral microdialysis
CPP: Cerebral perfusion pressure
DCI: Delayed cerebral infarction
ICP: Intracranial pressure
LPR: Lactate-to-pyruvate ratio
mBFV: Mean blood flow velocity
mRS: Modified Rankin Scale
SPM: Statistical parametric mapping
TCD: Transcranial color coded duplex sonography
